# A randomised, open-label, cross-over clinical study to evaluate the pharmacokinetic profiles of cigarettes and e-cigarettes with nicotine salt formulations in US adult smokers

**DOI:** 10.1007/s11739-019-02025-3

**Published:** 2019-02-02

**Authors:** Grant O’Connell, John D. Pritchard, Chris Prue, Joseph Thompson, Thomas Verron, Donald Graff, Tanvir Walele

**Affiliations:** 1 Imperial Brands plc, 121 Winterstoke Road, Bristol, BS3 2LL UK; 2 Celerion Inc., 621 Rose Street, Lincoln, NE 68502 USA

**Keywords:** Conventional cigarette, Electronic cigarette, E-cigarette, Pharmacokinetics, Nicotine delivery, Nicotine salt, Nicotine lactate

## Abstract

E-cigarettes containing ‘nicotine salts’ aim to increase smoker’s satisfaction by improving blood nicotine delivery and other sensory properties. Here, we evaluated the pharmacokinetic profiles and subjective effects of nicotine from two e-cigarette device platforms with varying concentrations of nicotine lactate (nicotine salt) e-liquid relative to conventional cigarettes. A randomised, open-label, cross-over clinical study was conducted in 15 healthy US adult smokers. Five different e-cigarette products were evaluated consecutively on different days after use of own brand conventional cigarette. Plasma nicotine pharmacokinetics, subjective effects, and tolerability were assessed following controlled use of the products. The rate of nicotine absorption into the bloodstream was comparable from all e-cigarettes tested and was as rapid as that for conventional cigarette. However, in all cases, nicotine delivery did not exceed that of the conventional cigarette. The pharmacokinetic profiles of nicotine salt emissions were also dependent upon the properties of the e-cigarette device. Subjective scores were numerically highest after smoking a conventional cigarette followed by the *my*blu 40-mg nicotine salt formulation. The rise in nicotine blood levels following use of all the tested e-cigarettes was quantified as ‘a little’ to ‘modestly’ satisfying at relieving the desire to smoke. All products were well tolerated with no notable adverse events reported. These results demonstrate that, while delivering less nicotine than a conventional cigarette, the use of nicotine salts in e-cigarettes enables cigarette-like pulmonary delivery of nicotine that reduces desire to smoke.

## Introduction

According to Public Health England and the Royal College of Physicians, electronic cigarettes (e-cigarettes) are likely to be at least 95% less harmful than conventional cigarettes [1, 2]. This view was recently reaffirmed, with a further comment from Public Health England that e-cigarettes pose only a fraction of the harms that smoking does, and that smokers should be encouraged to switch [3]. Continuing to recognise that complete cessation of all tobacco and nicotine use as the best action smokers can take to improve their health, Public Health England and the Royal College of Physicians are clear that encouraging and assisting smokers who are neither interested nor willing to quit smoking to switch to using nicotine products that are substantially less harmful than inhaled tobacco smoke is the next best option to help stop smoking [2, 3].

A growing body of evidence suggests that e-cigarettes can be an effective tool in helping smokers to quit smoking [3–6]. E-cigarettes have become the most common quitting aid for smokers in England, a finding supported by recent data, suggesting that 38.2% of smokers in the last quarter of 2017 reported using an e-cigarette in their recent quit attempt compared with 18% using nicotine replacement therapy (NRT) and 2.8% using Varenicline [3]. Studies investigating abstinence rates have found that e-cigarettes are helpful in enabling smokers to switch and subsequently remain smoke free. For example, a 2016 report estimated that 2.5% of smokers in England who used an e-cigarette in their quit attempt (22,000 people) succeeded where they would have failed if they had used nothing or a licensed nicotine product purchased over the counter [7]. An analysis of the data from the Eurobarometer 429 cross-sectional survey, performed in a representative sample of the European Union 28 Member States in 2014, found that smoking cessation with daily use of e-cigarettes was over 47% [8]. A recent study by Manzoli and colleagues followed up 236 e-cigarette users (all of whom were ex-smokers), 491 smokers, and 232 dual users for 12 months. They reported that 61.9% of the vapers were still abstinent from tobacco smoking after 12 months, compared with just 20.6% of the smokers and 22.0% of the dual users, suggesting that e-cigarettes can be effective in helping smokers abstain [9]. Research undertaken by Polosa and colleagues has also shown that the provision of e-cigarettes to smokers that have expressed no prior commitment to stopping smoking is associated with a significant reduction in smoking prevalence [10].

While there is a growing consensus that e-cigarettes are substantially less harmful than smoking and have the potential to generate substantial public health benefits at a population level if significant numbers of smokers switch from smoking to e-cigarette use [2, 3, 11, 12]; at this time, only minority of smokers have fully switched to vaping. For example, in the UK, there are an estimated 7.4 million adults who continue to smoke [13]. Whilst inaccurate beliefs on the relative harmfulness of e-cigarettes may be deterring many smokers from even trying an e-cigarette [3], it also suggests that currently available e-cigarettes do not provide smokers with the sensory experience they require from their conventional cigarettes [14]. Given the US Food and Drug Administration (FDA) have highlighted the role of nicotine in tobacco products, we hypothesise that the nicotine delivery profile of e-cigarettes may play a major role in consumer-reported satisfaction [15].

Adapting the speed of nicotine delivery from e-cigarettes may assist smokers in fully switching [16]. Nicotine replacement therapies (NRT) such as nicotine gums, patches and inhalators, deliver nicotine much more slowly and at lower doses than conventional cigarettes [3, 15, 17]. In addition to the absence of the behavioural and sensorial aspects of the smoking experience using such products, this may explain the limited success rate of NRT in smoking cessation. Smoking abstinence using NRTs is reportedly less than 7% after 12 months [17]. There have been number of attempts to develop an inhaled product which would deliver nicotine through the lung and mimic the physiological response from smoking. However, many of them produced intolerable aversive reactions or delivered an ineffective dose of nicotine [18]. By contrast, as e-cigarettes have evolved through several technical innovations, their nicotine delivery has also improved, although it has been shown to vary considerably across e-cigarette products [19–21]. Under the same puffing regime, experienced e-cigarette users can achieve greater increases in blood nicotine levels than naïve users, albeit at a much slower rate than achieved by smoking a conventional cigarette [20, 22]. E-cigarette nicotine intake and delivery have also been shown to be related to, and influenced by, user puffing topographies [1, 2, 23, 24], which differ significantly from puffing behaviours associated with smoking [25, 26]. When focussing on experienced e-cigarette users, it has been shown that comparable or higher blood nicotine levels can be obtained compared to smoking [27, 28]. A recent pharmacokinetic study also found that similar doses and speed of nicotine delivery to conventional cigarettes can be achieved among users of more modern advanced tank e-cigarette devices [29].

The form of nicotine ordinarily used in e-liquids is termed as ‘freebase’ nicotine. Freebase nicotine is volatile. As a result, when an e-cigarette aerosol is inhaled by a user, the nicotine is more likely to off-gas from the aerosol droplets and deposit in the mouth/upper respiratory tract, where it is absorbed into the blood. Absorption in the oral cavity/upper respiratory tract is slower than that with conventional cigarette with pharmacokinetic studies indicating a profile which more closely resembles NRT than a conventional cigarette [18]. The need for more effective and appealing e-cigarette products to provide satisfying alternatives to smoking has led to the recent development and marketing of e-liquids containing ‘nicotine salts’. Nicotine salts are formed by the reaction of nicotine with a suitable acid and are less volatile than freebase nicotine [15]. As a result, a greater fraction of the nicotine in the salt form would be expected to remain in inhaled aerosol droplets until the aerosol reaches the alveoli for pulmonary absorption. For pulmonary absorption, once deposited, nicotine salts must first dissociate into freebase and acid, to enable the non-polar, lipid-soluble freebase nicotine to gain cellular entry at the alveoli [15]. If nicotine salts can more closely replicate cigarette-like nicotine delivery in the lung, they should enable switching to e-cigarettes, therefore helping to further realise the harm reduction potential of these products.

Currently, there are limited data in the published literature available on the pharmacokinetic profiles of e-cigarettes containing nicotine salts. Here we performed a randomised, open-label, six-period cross-over clinical study to evaluate the nicotine uptake from two e-cigarette device platforms with varying concentrations of nicotine lactate (nicotine salt) or freebase nicotine e-liquids relative to conventional cigarettes in US adult smokers. In addition, subjective effects and tolerability of the tested products were also assessed.

## Methods

### Study design

This randomised, open-label, six-period crossover study (ClinicalTrials.gov; NCT03822546) was approved by the Institutional Review Board of Chesapeake Research Review (Maryland, USA) and was conducted in accordance with the International Conference on Harmonisation (ICH) Harmonised Tripartite Guideline for Good Clinical Practice (GCP) and the Declaration of Helsinki. Up to 15 adult subjects (at least six subjects of each sex) participated in this study which was performed at a single clinical site (Celerion, Nebraska, USA) in a confined setting over 6 days in April 2018. All subjects provided written informed consent prior to the study start.

### Participants

Fifteen healthy American smokers aged 21–65 years were eligible for the study if they had smoked ≥ 10 manufactured cigarettes (no restrictions on brand type) per day for at least the last year. Women of child-bearing potential were eligible only if they were using an accepted method of contraception. All subjects had an expired carbon monoxide level of > 10 ppm at screening and tested positive for urinary cotinine (≥ 500 ng/mL). Subjects could try each product after check-in on Day 1 to ensure that they would be willing to use the products during the pharmacokinetic evaluations. In total, subjects abstained from using any tobacco or nicotine-containing product for at least 18 h prior to use on Day 1 (conventional cigarettes), and thereafter were provided products at 24-h intervals.

Participants were excluded if they had a known or suspected hypersensitivity to any component of the e-liquid formulations; were taking or receiving prescription smoking cessation medicines; were willing or considering to stop smoking; had a history or presence of clinically significant pulmonary, cardiovascular, renal, hepatic, neurological, haematological, endocrine, oncological, immunological or psychiatric condition that could place them at risk or interfere with the interpretation of the study data; were a self-reported ‘puffer’, i.e., smokers who draw smoke into their mouth and throat but do not inhale; or had a body mass index (BMI) of less than 18 kg/m^2^ or greater than 40 kg/m^2^. Women who were breastfeeding were excluded from the study.

No subjects reported previous e-cigarette use prior to screening for the study and, thus, are considered naïve users.

### Investigational products

The five e-cigarette products tested were (1) *my*blu pod-system containing 25-mg nicotine (‘freebase’) tobacco flavour; (2) *my*blu pod-system containing 16-mg nicotine lactate tobacco flavour; (3) *my*blu pod-system containing 25-mg nicotine lactate tobacco flavour; (4) *my*blu pod-system containing 40-mg nicotine lactate tobacco flavour; and (5) blu PRO open system containing 48-mg nicotine lactate tobacco flavour. The reference cigarettes, provided by the subjects, were their preferred brand of commercially available conventional cigarette.

The e-cigarette products assessed in this study were obtained from the US market and are manufactured by Fontem Ventures B.V. (The Netherlands). The *my*blu device is a rechargeable, closed pod-system e-cigarette, consisting of two segments. A rechargeable battery section (battery capacity, 350 mAh) and a replaceable e-liquid containing pod (volume, 1.5 mL; coil resistance, 1.3 Ω). The *my*blu device delivers on average 7–8 mg of aerosol per puff under machine vaping conditions [30]. The blu PRO device is a rechargeable, open-system e-cigarette, consisting of two segments. A rechargeable battery section (battery capacity, 1100 mAh) and a refillable clearomiser (volume, 2.0 mL; coil resistance, 1.8 Ω). The blu PRO device delivers on average 2–3 mg of aerosol per puff.

### Procedure

Subjects visited the study site for a screening visit within 28 days prior to baseline Day 1. Screening evaluations included physical examination, vital signs, ECG, clinical laboratory tests (clinical chemistry, haematology, urinalysis, and serology), urine drug, cotinine, and alcohol screen, and serum pregnancy tests for females only. On Day 1, subjects completed a trial of all investigational products and completed the Fagerström Test for Cigarette Dependence (FTCD). On Days 1–6, after overnight smoking and nicotine abstinence, participants used the assigned product under controlled conditions according to their randomisation sequence. On Day 1, all participants smoked a single preferred brand of conventional cigarette (not randomised) with puffs taken at 30-s intervals. On each of Days 2–6, participants received their randomised assigned e-cigarette with a fully charged battery and fresh pre-filled pod (*my*blu) or clearomiser (blu PRO) and used the product for 10 inhalations every 30 s for 3 s in duration. All product use sessions were directly monitored by the clinic staff, who indicated to the subjects when to start and stop puffing. All e-cigarette products were weighed before and after use to determine the quantity of e-liquid consumed.

### Study assessments

#### Pharmacokinetic analysis

Plasma nicotine pharmacokinetic assessment was the primary outcome measure for this study. On each study day (Days 1–6), 4 mL of whole blood was collected 5 min prior to and at 2, 3, 4, 5, 6, 7, 8, 9, 10, 12, 15, and 30 min following the start of product use. Plasma was separated by centrifugation within 60 min of collection, aliquoted and stored at − 20 °C. The determination of plasma nicotine concentrations was carried out using a validated LC–MS/MS method, over a calibration range spanning from the lower limit of quantification of 0.200–25.0 ng/mL.

The pharmacokinetic parameters determined were the mean maximum plasma nicotine concentration (*C*_max_), the median time to maximum plasma nicotine concentration (*T*_max_), and the mean area under the plasma nicotine concentration–time curve, from time 0 to 30 min (AUC_0–30_).

Pharmacokinetic analyses were performed using Phoenix™ WinNonlin^®^ Version 7.0.

#### Subjective effects

To assess the impact of the investigational products on desire to smoke, their effects on aspects of nicotine satisfaction and other subjective measures, responses were elicited on a Likert-type scale with responses ranging from 1 (not at all) to 7 (extremely). The following questions were compiled by the clinical research organisation and were based on previous questionnaires designed to assess the effects of tobacco product use [31, 32]: Did it make you dizzy? Did it make you nauseous? Did you enjoy it? Did it relieve the urge to smoke? Was it enough nicotine? Was it too much nicotine? This assessment was made 20 min after the start of product use.

#### Safety and tolerability

Safety and tolerability were assessed by the study investigator. The incidence and nature of any adverse events (AEs) and concomitant medications throughout the study were recorded by assessment of reported events, physical examination, monitoring of vital signs (respiratory rate, heart rate, blood pressure, ECG, and temperature) and clinical biochemistry tests (clinical chemistry, haematology and urinalysis).

### Statistical analyses

Statistical summarizations were performed using SAS^®^ Version 9.3.

The sample size was determined adequate for nicotine bioavailability comparisons and was selected based on similar pharmacokinetic studies on e-cigarette products [20–22, 31–34].

Participants were included in the pharmacokinetic population if they completed use of the tested investigational product and evaluable data for the specified endpoints were obtained. Baseline adjustments were performed. For the subjective effects, descriptive statistics were calculated. For AEs, investigational product use-emergent AEs are summarised; an investigational product use-emergent AE was defined as an AE that started or worsened at the time of, or after, the first investigational product is used.

To determine whether pharmacokinetic parameters following use of the e-cigarette products were significantly different from those of the conventional cigarette, a multiple test comparison with a *P* value adjustment based on the Westfall–Young approach was used. If the *P* value was less than 0.05, the difference was considered to be statistically significant where *P < 0.05, **P < 0.01 and ***P < 0.001.

Individual participants’ subjective scores to each investigational product type were analysed by means of a non-parametric randomised block analysis of variance Friedman test. Where a significant difference was observed (*P < 0.05), post hoc comparisons were performed using a Nemenyi test, with differences considered as statistically significant for *P < 0.05.

## Results

### Study population

Fifteen subjects were enrolled to test each investigational product. There was one non-completer for the *my*blu 25 mg (freebase) and blu PRO 48 mg (nicotine lactate) and two for the *my*blu 25 mg (nicotine lactate) which did not result from product-related AEs. The baseline characteristics of the subjects are summarised in Table 1. No subjects reported previous e-cigarette use prior to screening for the study.

**Table 1:** Demographic and baseline characteristics of the pharmacokinetic population

Variable	Characteristics
Number of subjects, *n*	15^a^
Smoker type	10 ‘full flavour’ cigarettes; 5 ‘light’ cigarettes
	1 menthol
	14 non-menthol
Age (years)	
Mean (SD)	42.3 (12.41)
Range	24–62
Sex, *n* (%)	
Male	9 (60%)
Female	6 (40%)
BMI (kg/m^2^)	
Mean (SD)	28.137 (5.1412)
Range	20.20–39.49
FTCD (total score)	
Mean (SD)	5.5
Range	3–9

*BMI* body mass index; *FTCD* Fagerström Test for Cigarette Dependence Questionnaire; *SD* standard deviation

^a^15 enrolled, 1 participant did not use *my*blu 25 mg (freebase) and blu PRO 48 mg (nicotine lactate) and 2 participants did not use *my*blu 25 mg (nicotine lactate)

### Study product use

The mean number of puffs taken during each product use session and the change in e-cigarette mass are reported in Table 2. Ten puffs were taken from each of the *my*blu products containing nicotine lactate during each use session, while one puff from the *my*blu 25 mg (freebase), blu PRO 48 mg (nicotine lactate), and conventional cigarette was missed by two subjects. All conventional cigarettes were consumed in 9 or 10 puffs. Mean product mass changes from pre- to post-use was greatest for the *my*blu 16-mg (nicotine lactate) product, followed by the *my*blu 40 mg (nicotine lactate), *my*blu 25 mg (nicotine lactate), *my*blu 25 mg (freebase), and blu PRO 48 mg (nicotine lactate), respectively.

**Table 2:** Summary of product use by investigational product type

	Conventional cigarette	*my*blu 40 mg (nicotine lactate)	*my*blu 25 mg (nicotine lactate)	*my*blu 16 mg (nicotine lactate)	blu PRO 48 mg (nicotine lactate)	*my*blu 25 mg (freebase)
Number of puffs	9.9 (0.35)	10.0 (0.00)	10.0 (0.00)	10.0 (0.00)	9.9 (0.27)	9.9 (0.27)
Product mass change (g)	NA	0.04853 (0.022660)	0.04425 (0.018735)	0.06526 (0.028930)	0.01791 (0.013702)	0.04396 (0.019524)

All values are arithmetic mean and standard deviation (SD)

*NA* not applicable

### Pharmacokinetic analysis

**Fig. 1:**
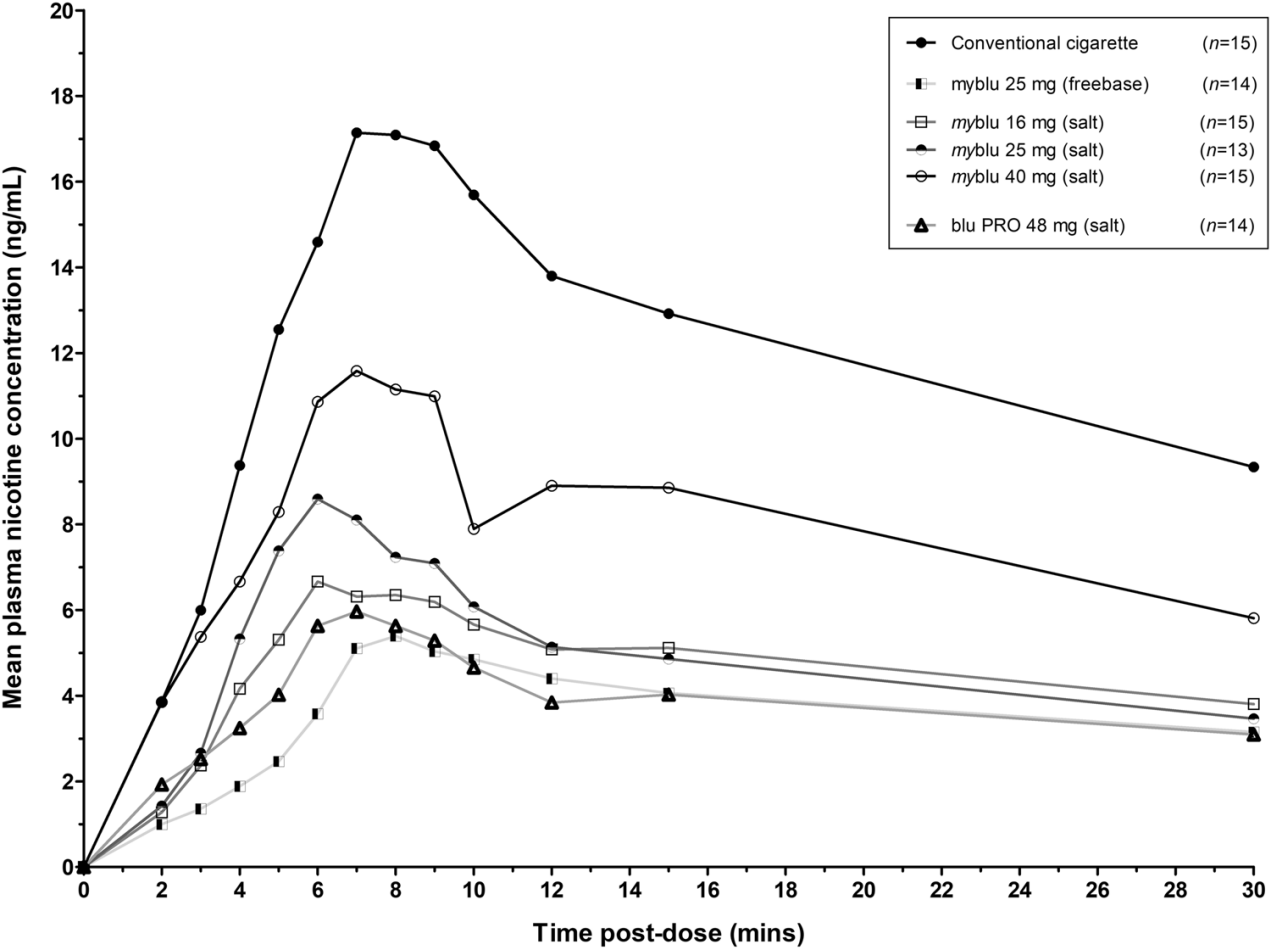
Pharmacokinetic profiles: mean plasma nicotine concentration by investigational product (linear scale) over 30 min

The mean plasma nicotine concentration profiles of the conventional cigarettes and the investigational e-cigarette products are shown in Fig. 1 and the pharmacokinetic parameters for each product are reported in Table 3. The *my*blu 40 mg (nicotine lactate) product had the closest set of pharmacokinetic parameters to that of the conventional cigarette.

**Table 3:** Summary of pharmacokinetic parameters by investigational product type

	Conventional cigarette	*my*blu 40 mg (nicotine lactate)	*my*blu 25 mg (nicotine lactate)	*my*blu 16 mg (nicotine lactate)	blu PRO 48 mg (nicotine lactate)	*my*blu 25 mg (freebase)
*C*_max_, ng/mL	17.81 (49.6)	10.27 (83.6)	7.58 (80.6)^**^	6.51 (76.5)^***^	4.85 (108.3^)***, †^	5.048 (49.9)^***^
*T*_max_, median (range), min	8.05 (5.00–15.13)	7.9 (1.97–15.0)	6.03 (4.58–16.77)	6.967 (3.98–15.05)	6.908 (2.35–15.03)	8.034 (2.28–15.10)
AUC_0–30_, ng*min/mL	324.9 (35.8)	190.7 (71.8)	125.2 (53.4)^***^	118.5 (60.8)^***^	84.84 (89.8)^***, ††^	98.99 (35.8)^***^

All values are geometric mean and geometric coefficient of variation (CV %) unless stated otherwise

*C*_max_ maximum plasma nicotine concentration; *T*_max_ time to maximum nicotine concentration; AUC_0–30_ area under the concentration–time curve from time zero to the last quantifiable concentration (30 min)

*Significant difference compared to conventional cigarette (**P < 0.01 and ***P < 0.001)

^†^Significant difference between *my*blu 40 mg (nicotine lactate) and blu PRO 48 mg (nicotine lactate) (^†^*P* < 0.05; ^††^*P* < 0.01)

All e-cigarette products had a median *T*_max_ that was in the range of the conventional cigarette indicating aerosol deposition in the deep lung facilitating rapid nicotine absorption from all tested e-cigarettes.

The *C*_max_ for all the e-cigarette products was significantly lower (*P *< 0.05) than that of the conventional cigarette, except for the *my*blu 40-mg (nicotine lactate) product which was not significantly different. An increase in the nicotine lactate concentration in the *my*blu device also resulted in an increased *C*_max_, with a trend toward dose proportionality. Of note, the *C*_max_ was significantly higher (*P *< 0.05) for the *my*blu 40-mg (nicotine lactate) product than the blu PRO 48-mg (nicotine lactate) open-system device which is likely to be reflective of the technological improvements of the newer *my*blu device.

For all e-cigarette products, the extent of nicotine absorption (AUC_0–30_) was significantly less (*P *< 0.05) than that of the conventional cigarette, except for the *my*blu 40-mg (nicotine lactate) product which was not significantly different. With increasing nicotine lactate concentrations in the *my*blu device, the AUC_0–30_ increased, with a trend toward dose proportionality. The blu 48-mg (nicotine lactate) product had an AUC_0–30_ significantly less than that of the *my*blu 40-mg (nicotine lactate) product again indicating the improved aerosol generation properties and nicotine delivery of the *my*blu device vs the blu PRO device, which uses an older aerosol generation technology.

### Subjective effects

Subjective effect scores are reported in Table 4. Subjective scores were numerically highest for all questions after use of the conventional cigarette followed by the *my*blu 40-mg (nicotine lactate) product. For three questions (Did it make you dizzy? Did it relieve the urge to smoke? and Was it enough nicotine?), a significant difference was observed between the six investigational products (*P *< 0.05). In general, a rapid absorption of nicotine with a higher *C*_max_ appears to produce greater relief in desire to smoke which may be important for facilitating smoker switching and preventing relapse. The other subjective measures appear to be numerically comparable and not significantly different across all formulations irrespective of nicotine delivery, indicating that in addition to nicotine, other behavioural and sensorial elements that e-cigarettes provide play a role in satisfaction.

**Table 4:** Summary of subjective evaluations of each investigational product type

	Conventional cigarette	*my*blu 40 mg (nicotine lactate)	*my*blu 25 mg (nicotine lactate)	*my*blu 16 mg (nicotine lactate)	blu PRO 48 mg (nicotine lactate)	*my*blu 25 mg (freebase)
Did it make you dizzy?*	3.7 (1.80)	2.8 (1.78)	2.1 (1.32)	1.5 (0.74)	1.7 (0.99)	1.9 (1.73)
Did it make you nauseous?	1.9 (1.44)	1.4 (0.91)	1.2 (0.44)	1.1 (0.26)	1.4 (0.84)	1.3 (0.83)
Did you enjoy it?	4.9 (1.44)	4.0 (1.36)	3.5 (1.98)	3.5 (1.46)	3.2 (1.81)	3.5 (1.87)
Did it relieve the urge to smoke?*	5.5 (1.60)	4.1 (1.79)	3.5 (1.98)	3.3 (1.91)	3.1 (2.11)	3.6 (2.10)
Was it enough nicotine?*	5.4 (1.55)	4.3 (1.79)	3.1 (1.93)	3.3 (1.99)	3.2 (2.08)	4.0 (1.96)
Was it too much nicotine?	2.4 (1.55)	2.2 (1.66)	1.5 (0.97)	1.7 (1.11)	1.4 (0.63)	2.5 (2.21)

Scale: 1, not at all; 2, very little; 3, a little; 4, modestly; 5, a lot; 6, quite a lot; 7, extremely

All values are mean and standard deviation (SD)

* Significant difference between the six investigational products (*P < 0.05)

### Safety and tolerability

There were no serious adverse events reported during the study. Product-use AEs were infrequent with four subjects reporting 10 AEs in this study, none of which led to discontinuation. Vessel puncture site pain was the most frequently reported AE, experienced by two subjects. All remaining AEs were experienced by one subject each. All AEs were mild in severity, apart from moderate insomnia with the blu PRO 48 mg (nicotine lactate). The principal investigator considered one AE of headache with the *my*blu 25 mg (freebase) to be possibly related to study product and the remaining nine events unlikely or unrelated.

The use of the e-cigarette products under the study conditions appeared to be well tolerated by the healthy adult smokers in this study.

## Discussion

This study provides new insights into e-cigarette products that contain nicotine lactate when used by adult smokers under controlled conditions. The data indicate that nicotine in lactate salt formulation is rapidly delivered into the systemic circulation following inhalation using nicotine lactate e-cigarette products with plasma pharmacokinetic profiles consistent with pulmonary absorption; however, with nicotine doses less than that of a conventional cigarette. Compared to conventional cigarettes, exposure to nicotine was 41% lower following use of *my*blu 40 mg (nicotine lactate), 60% lower following use of *my*blu 25 mg (nicotine lactate), 63% lower following use of *my*blu 16 mg (nicotine lactate), and 70% lower following use of *my*blu 25 mg (freebase). The blu PRO 48-mg (nicotine lactate) product delivered approximately 73% less nicotine compared to conventional cigarettes.

As might be expected, variations in the concentration of nicotine content of the e-liquid also had an impact on nicotine delivery. Indeed, the pharmacokinetic profile of the 40-mg nicotine lactate e-cigarette product was approaching that of a conventional cigarette; whereas, the 16-mg comparator was significantly different. The subjective data also show that the 40-mg nicotine lactate product reduced the desire to smoke numerically more than the 16-mg nicotine lactate product; this may be consistent with an efficient transfer of more nicotine to the lungs and a rapid rise of nicotine absorption in the plasma. These initial findings suggest that e-cigarettes with a higher concentration of nicotine in nicotine lactate form may be more effective and more appealing products for adult smokers switching from cigarettes to vapour products. This is in line with public health recommendations in the UK and elsewhere, e.g. [3, 12, 35].

From smoking a conventional cigarette, a plasma nicotine concentration of around 4 ng/mL has been reported to occupy up to 90% of available α4β2* nicotinic acetylcholine receptors in the brain and significantly reducing desire to smoke [36]. By contrast, following use of a licensed nicotine inhaler, a peak receptor occupancy of 60% is only reached after 3 h which was insufficient to reduce desire to smoke [37]. Thus, it appears that a shorter *T*_max_ is important to activate most nicotine receptors and provide smoker satisfaction from alternative products. In our study, the *T*_max_ values for all e-cigarettes were in a range which is comparable to published conventional cigarette data [38]. A study is underway to assess smoking reduction and switch rates associated with nicotine lactate e-cigarette ad libitum use in the real-world with medicinal nicotine replacement product and freebase e-cigarette comparators.

The European Union Tobacco Products Directive (EUTPD) mandates that the maximum nicotine content of an e-liquid cannot exceed 20 mg/mL. Recent research has shown that the use of lower nicotine concentration e-liquids may be associated with ‘compensatory behaviour’ as e-cigarette users puff more deeply, more frequently and for longer to obtain a level of nicotine that reduces desire to smoke [39]. In the present study, the *my*blu 40-mg nicotine lactate product had the closest nicotine uptake profile to the conventional cigarette with the greatest relief in desire to smoke. However, this product would not be permitted in the European Union. Both Public Health England and the Royal College of Physicians have stated that the cap on nicotine concentrations imposed by the EUTPD may limit the effectiveness of e-cigarettes as a smoking substitute, particularly for heavier smokers [1, 2]. Based on our initial data presented here, and other research insights [40], the EUTPD nicotine concentration limit should be reviewed in line with the scientific literature to ensure that adult smokers have ready access to better alternatives that reduce desire to smoke. Higher nicotine strength liquids with nicotine lactate formulations and suitable flavour options that can be marketed to smokers may maximise the public health potential of e-cigarettes.

This was a small, short-duration study that was not designed to fully evaluate safety. However, reports of AEs were recorded, and the study was conducted to GCP. Use of the nicotine lactate e-cigarettes was well tolerated by the participants with no severe or serious AEs and no participants discontinued the study owing to an AE.

The main limitation of this study is that the e-cigarette puff profile was fixed (use was not ad libitum) to obtain clear pharmacokinetic profiles and blood sampling was collected for only 30 min. A more robust pharmacokinetic assessment is planned in future studies. It is likely that smokers using the e-cigarette devices in the ‘real world’ would change their behaviour to adapt to the new products and own preference. To assess the amount of nicotine and smoker satisfaction that nicotine lactate e-cigarettes provide under real-world conditions, it would be beneficial to conduct a long-term study that allows subjects to adapt their behaviour to product use. Another limitation is that study participants were not experienced e-cigarette users. However, prior to study start, subjects could use each of the products, although their preferences on the products ahead of the study start were not known. This may have influenced the reporting of subjective effects. The e-cigarette products may not have been used in an optimal way or in the same way they would have been used had the participants been familiar with the product; a longer familiarisation training period would be beneficial in future studies. Furthermore, only tobacco-flavoured e-cigarettes were assessed in this study; had the participants the opportunity to vary the flavour it may have influenced product use and satisfaction. In the present study, only nicotine lactate salt formulations were assessed; further research is also warranted to determine the pharmacokinetic profiles and efficacy of other nicotine salt formulations.

## Conclusion

In summary, the results of this study indicate that the use of nicotine lactate in e-cigarettes has promise as an effective form of nicotine replacement. The pharmacokinetic and subjective data demonstrate that nicotine lactate can be used to deliver nicotine via the pulmonary route for increased speed of absorption, albeit with a maximum nicotine level that did not exceed the conventional cigarette, coupled with acceptable subjective satisfaction and relief of desire to smoke. Further studies are warranted to fully assess the efficacy of nicotine lactate in aiding smokers to fully switch to e-cigarettes in the real-world, as well as the research on the role of flavours and other innovations that can maximise the public health potential of alternatives to cigarettes for adult smokers.
